# State, food, and me: an autoethnographic reflection on the sociocultural dimensions of Chinese women’s eating disorders

**DOI:** 10.1186/s40337-025-01498-2

**Published:** 2025-12-13

**Authors:** Yueying Li

**Affiliations:** London School of Economics and Political Science, London, UK

**Keywords:** Eating disorders, China, Autoethnography, Feminism, OSFED

## Abstract

**Background:**

Eating disorders among Chinese women have been growing in recent years, yet, little research has explored these experiences in non-Western contexts. This study aimed to challenge the dominant Western-centric models, which often universalise psychiatric frameworks and overlook culturally embedded aspects of distress. It critically examines how sociocultural forces in contemporary China shape the development and recovery of eating disorders among Chinese women.

**Method:**

This paper adopted a critical autoethnographic approach, grounded in the author’s decade-long lived experience as a Chinese woman with eating disorders.

**Results:**

The paper highlighted key sociocultural barriers faced by Chinese women, including the societal emphasis on women’s appearance, pervasive competition culture, and Confucian gender norms. It also identified specific structural challenges in China, such as inadequate psychiatric resources, treatment paradigms neglecting trauma, entrenched stigma surrounding mental illness, and a cultural history that moralises food consumption and waste.

**Conclusion:**

Guided by feminist and poststructuralist critiques, the paper argued that eating disorders among Chinese women are not merely personal or psychological conditions, but reflect broader social tensions linked to neoliberal governmentality, gender inequality, and moral values in contemporary China. Recovery, therefore, cannot be reduced to clinical intervention alone; it also demands reclaiming subjectivity beyond sociocultural constraints.

## Abstract (plain language summary)

This paper adopted an autoethnographic approach to explore how sociocultural factors in China influence the development and recovery of eating disorders among women. Drawing upon the author’s decade-long experience of living with eating disorders, it highlighted the pressures Chinese women face, including the emphasis on beauty, social competition, and traditional gender expectations. The paper also discussed challenges in the mental health system, including lack of resources and stigma. The author showed that eating disorders are not just individual or medical problems, but deeply connected to broader forces of neoliberal governmentality, gender inequality, and moral values. This paper aimed to offer insights for clinicians, educators, and policymakers, and suggested that recovery requires not only medical care but also a deeper psychological and cultural transformation.

## Introduction

Writing this paper has been a deeply unsettling process. This unease stems, in part, from the fact that being someone who has lived with eating disorders for 10 years, much of my waking life remains enveloped in tension. It also arises from my awareness — as a perfectionistic researcher — that the epistemological reach of this critical autoethnography may be limited. After all, it is a methodology that relies on the self as both subject and object, generating knowledge through sustained personal reflection. I am also conscious of the possibility that my identity may shape my analysis in ways that are not universally resonant. As Crenshaw’s concept of intersectionality reminds us, the meanings of social experiences are always filtered through intersecting axes of identity [1]. My narrative, therefore, may not speak for all Chinese women who suffer from eating disorders. Still, I choose to write. I choose to strip back the scab and gaze directly at the wound, because only through chewing on the pain can I begin to understand it.

Over the course of my illness, I received treatment in both Shanghai and Oxford. These cross-cultural experiences have allowed me to compare different paradigms of treatment and to reflect critically on the sociocultural challenges specific to Chinese women with eating disorders. At a time when eating disorders are increasing across all age groups in China, I hope that this critical autoethnography, through the specificity of lived experience, can help address the scarcity of sociocultural research on Chinese eating disorder population and challenge dominant Western-centric perspectives on eating disorders.

This paper aims to investigate how sociocultural factors influence both the development and recovery of eating disorders among Chinese women. I begin with a review of the relevant literature, followed by an explanation of the critical autoethnographic method. Then, I present an analysis of key sociocultural factors implicated in Chinese women’s disordered eating, and a reflexive discussion on what eating disorders reveal about broader issues in contemporary Chinese society. Finally, I conclude by summarising the main insights and offering practical suggestions for those on the path to recovery.

## Literature review

The American Psychiatric Association defines eating disorders as “behavioral conditions characterized by severe and persistent disturbances in eating behaviors and associated distressing thoughts and emotions” [2]. Individuals with eating disorders typically experience persistent preoccupations and anxiety surrounding food, weight, and body shape, and may engage in behaviours such as “restrictive eating or avoidance of certain foods, binge eating, purging by vomiting or laxative misuse or compulsive exercise” [2]. The most widely used diagnostic reference is the *Diagnostic and Statistical Manual of Mental Disorders* (DSM), whose latest version, *DSM-5-TR*, categorises eating disorders into several forms, including anorexia nervosa (AN), bulimia nervosa (BN), binge eating disorder (BED), avoidant restrictive food intake disorder, other specified feeding and eating disorder (OSFED), and pica and rumination disorder [2]. Among these, AN, BN, and BED are the most prevalent [3], with women significantly overrepresented [4].

The diagnosis of eating disorders is often unstable and subject to change, as individuals may shift between different types over time [5]. I resonate deeply with this diagnostic fluidity. At age 17, I was diagnosed with AN due to my extremely low weight. As I regained weight but lost control over food, I was reclassified as having BED. Later, purging behaviours led to a diagnosis of BN. Most recently, I was diagnosed with atypical anorexia nervosa — a subtype under OSFED, since my weight had returned to a normal range, yet I continued to restrict my food intake in daytime and occasionally engaged in bingeing and purging at night. Personally, I do not attach much importance to these shifting labels; they all mark forms of suffering that haunt me daily. While I recognise that diagnosis can guide treatment, I also question whether standard medical categories can truly capture the lived complexity of these experiences.

### Etiology of eating disorders

Although the etiology of eating disorders remains unclear, it is widely accepted that genetic and neurobiological factors play a role, while psychological traits and sociocultural environments act as catalysts [6]. Medical studies have found that individuals with a family history of eating disorders or other mental health conditions are at higher risk [7]. Fairburn, the founder of Enhanced Cognitive Behavioural Therapy (CBT-E), also notes that dieting, one which is “so much more common among women than men. greatly increases the risk of developing eating problems” [8]. Research suggests that fluctuations of more than 10% in adolescent girls’ body weight increase their risk of developing eating disorders by seven times [9].

Psychologists have identified certain personality-related traits and psychological risk factors that may predispose individuals to eating disorders. These include perfectionism, cognitive inflexibility, emotional dysregulation, avoidance motivation, body image dissatisfaction, and low self-esteem [10–12].

As recognition grows that eating disorders cannot be fully explained through medical or psychological models alone, sociocultural factors have attracted increasing scholarly attention. These include weight-based bullying, media reinforcement of thin ideals, and maladjustment in cultural changes [7, 13, 14]. Less discussed, but equally important, is the role of food system transformations: the simplification of modern food preparation, according to Pirie, is a necessary condition for the rise of eating disorders [4].

Given the salient gender disparity among individuals with eating disorders, feminist scholars have sought to wrest eating disorders away from the pathologising appearance-based interpretations [15, 16]. They argue that patriarchy is a central force behind women’s struggles with food and body. Not only does the male gaze undermine women’s subjectivity, but patriarchal norms also constrain women’s access to power within both family and society [15]. Consequently, eating disorders may be interpreted as embodied responses to systemic gender oppression. Although contemporary shifts have afforded women greater legal and economic rights, the ambiguities around women’s role often produce an identity conflict, as women are simultaneously expected to assert independence while conforming to men’s beauty standards. Kilbourne mentioned that women are more likely to internalize social failure and turn to food for emotional compensation [17], as a result, the diet industry’s messaging around slenderness exacerbates their vulnerability to eating disorders [4, 18, 19].

### Semiology of eating disorders

Sociocultural perspectives have inspired feminist and poststructuralist scholars to explore the semiology of eating disorders, specifically, how disordered eating behaviours serve as symbols of psychological and relational tensions. In childhood, food is often a site of negotiation between love and control. As Pirie observes, “it was through food that parents expressed love and exercised control… denying consumptive rights to disobedient children” [4]. From this view, a refusal to eat in juvenile anorexia can be interpreted as a symbolic rebellion against parental authority.

Among adult women, eating problems often signify deeper “hungers for recognition, achievement, and encouragement” [15]. Gordon argues that anorexia reflects a “desire to escape womanhood and achieve a type of masculine ideal” [20], while bulimia reflects an intense need for acceptance and an inordinate drive to please men. Each type of disordered eating thus embodies different meanings.

### Eating disorders in non-Western contexts

Because eating disorders have historically been conceptualized as a culture-bound illness of the West [13], most research has focused on white middle-class women in Euro-American contexts [15]. Transcultural scholars have criticised this bias and argued that both the etiology and semiology of eating disorders vary in non-Western societies.

For example, Lester notes that in Mexico, eating disorders cannot be simply understood through the lens of thwarted individuation, as Mexican familism emphasizes relational responsibility and embeddedness [21]. Hence, treatment there centers not on self-assertion, but on the regulation of emotional dynamics within relationships [21]. Similarly, Thompson found that for women of colour, food often serves as a survival strategy “in response to racism, homophobia, classism, the stress of acculturation, and emotional, physical, and sexual abuse” [15].

In China, eating disorders must also be interpreted through specific cultural logics. Based on interviews with 11 hospitalized female adolescents and young adults and their parents, Vu-Augier de Montgrémier et al. highlight how gender expectations, surveillance, and independence pressure shape disordered eating in Chinese female [19]. Holmes and Ma, drawing on interviews with 12 women diagnosed with BN and BED, argue that bingeing and purging can be seen as responses to contradictions in Chinese femininity, such as obeying family food rituals, navigating conflicting messages about female appetite, and striving for a body that signifies self-restraint and discipline [22].

### Research gaps

Although the global prevalence of eating disorders has remained relatively stable [23], the incidence among Chinese individuals has risen sharply over the past two decades [13]. While the studies by Vu-Augier de Montgrémier et al. [19] and Holmes and Ma [22] provide critical insights, such research remains limited in scope and number.

I was, in fact, one of the participants in Vu-Augier de Montgrémier et al.’s study [19]. When I revisited their paper five years later, I was shocked by how my perspectives had changed. As Paquette and Raine argue, women’s narratives about their bodies and disordered eating are not static but fluctuate as “women encounter new experiences and re-interpret old ones” [24]. My disagreement with some of my earlier reflections does not mean the researchers misrepresented me; rather, their cross-sectional design could not capture the dynamism of my lived experience.

After reading a wide body of literature, I also became increasingly dissatisfied with the medicalization of eating disorders. Pirie argues that medical discourses often strip away agency and the complex social meanings of disordered eating, reducing them to individualized pathology [4].

In her autoethnography of anorexia, Holmes calls for scholars to engage in dialogue with women who have experienced disordered subjectivities [16]. Yet, in the Chinese context, such first-person scholarship remains rare. Highly reflective narratives, such as Holmes’s piece, are essential for deepening our understanding of eating disorders. They offer not only more accurate insight into the lived experience of illness but also the potential to inform more culturally sensitive and innovative interventions.

## Research question and methodology

Koski emphasised that “illness experience is influenced not only by individual circumstances, subjectivity, and interpersonal relationships but also by broader cultural and social structural forces” [25]. In light of the aforementioned research gaps, this study investigates the sociocultural factors that influence the development and recovery of eating disorders among Chinese women. In this sense, the paper may be read as both a dialogue with and a longitudinal supplement to the studies by Vu-Augier de Montgrémier et al. [19] and Holmes and Ma [22].

The types of eating disorders addressed in this paper include AN, BN, BED, and OSFED. As defined by the National Eating Disorder Association, OSFED refers to individuals who do not meet the strict diagnostic criteria for AN, BN, or BED [26]. In China, OSFED remains significantly under-recognised, partly because its symptoms often manifest at a sub-clinical level [4]. As a result, many Chinese individuals with OSFED either dismiss the need for medical care or fail to receive appropriate diagnoses in hospitals that primarily recognise only AN, BN, or BED. I choose to incorporate OSFED in this study because it remains a significant category of eating disorder — one that affects a wide range of Chinese women, including myself. Furthermore, given my own queer identity, I do not apply a strict cisnormative or heteronormative definition of “Chinese women” in this study. Instead, I use the term to refer broadly to individuals assigned female at birth, and who identify, in some way, with the sociocultural expectations placed upon Chinese women.

Holmes notes, “it is not unusual in feminist writing on the subject for the author to lay claim to personal experience of an eating disorder” [16]. Inspired by her autoethnographic work which integrates personal reflection with sociocultural critique [27], I adopt a critical autoethnographic approach guided by analytic reflection and rooted in feminist and poststructuralist inquiry. Critical autoethnography is particularly suitable for this research, as it is underpinned by deep reflexivity and introspection [28]. It not only transcends mere factual recounting by foregrounding how the researcher’s interpretations are filtered through specific positionality, but also facilitates a critical self-awareness of the researcher’s implication within systems of power [28].

The empirical basis of this paper draws upon my lived experience with eating disorders in both China and the UK. By narrating and analytically reflecting on these experiences through feminist and poststructuralist lenses, I seek to connect personal insight to self-identity, cultural norms, communication practices, shared values, and broader sociocultural and sociopolitical issues [29]. Through these accounts, I aim to offer a more situated understanding of Chinese women’s experience of disordered eating.

## My journey with eating disorders

Before examining the sociocultural factors that shape the development and recovery of eating disorders among Chinese women, I feel it is necessary to briefly recount my own history. This account forms the foundation of my following analyses and reflections. Writing about my experience is not easy. I hesitate to revisit those memories because they are like needles; each recollection pierces my heart with wounds I inflicted on myself, and each sting still lingers. I also feel ashamed. These memories, though mine, carry a deep personal stigma that I fear exposing to judgment. I have “wasted” so much time on this problem, and despite countless times of reflection, I have yet to overcome it.

I was born in China and received a typical disciplined Chinese education through middle school. I then attended a high school alone in New Zealand. Although I was within the normal weight range, my double chin became a frequent subject of ridicule among classmates. It made me feel deeply inferior. In my mind, the only way to make my face thinner was to lose weight, what I now see as the immediate trigger of my anorexia.

After completing my university entrance exams, my high school counsellor contacted my parents and advised them to bring me home for proper treatment. At the time, I had never heard that family-based treatment is recognised as a leading intervention for adolescent eating disorders [30], nor had my parents ever heard of anorexia. By 2016, while many Chinese people were aware of psychological issues like depression or anxiety, eating disorders remained obscure. I come from a small city in China, which had a local mental health center but no doctor specializing in eating disorders. My parents, like me, were completely uninformed. They assumed my condition resulted from dissatisfaction with Western food and believed everything would be fine once I returned home. During that break, they simply forced me to eat and did not seek psychiatric care. Although my weight increased, I became anxious and afraid that I couldn’t stop eating. Once my body returned to a “normal” weight, my parents dismissed my distress and declared me “healthy.” Lacking adequate knowledge, I, too, downplayed my condition and returned for university.

University life proved unbearable. I struggled to focus, haunted constantly by thoughts of food. I trembled when I ate, yet couldn’t suppress the urge to eat. I remember telling my mother about these symptoms, but she dismissed them as overthinking. Eventually, the psychological and physical pain became so overwhelming that I could no longer study. For the first time, I contemplated suicide. That moment finally prompted my parents to recognise the severity of my condition. I took a leave of absence and returned to China. My parents brought me to the Shanghai Mental Health Center — one of the only two hospitals in the country specializing in eating disorders. There, I was hospitalized for inpatient treatment.

Although the systematic care I received in hospital enabled me to return to university, I have not managed to free myself from the grip of this illness. Even now, I continue to be tormented by the anxiety and pain surrounding eating. It is from this unresolved space that I write — not as a recovered subject looking back, but as someone still struggling to understand what recovery might mean within and against the sociocultural conditions that first gave rise to this suffering.

## Sociocultural factors influencing eating disorders development

Before my undergraduate psychology professor recommended Fairburn’s *Overcoming Binge Eating: The Proven Program to Learn Why You Binge and How You Can Stop* [8], I believed my eating disorder was primarily rooted in biological and psychological causes. I once wondered whether my mother might also have had disordered eating, as she rarely ate dinner and engaged in extensive daily exercise. Still, I used to insist that my perfectionistic tendency was the main cause and largely blamed myself.

Reading Fairburn’s [8] book disrupted my earlier belief in a purely intrapsychic explanation. It opened up the soothing possibility for me that eating disorders might also be responses to external pressures. Reflecting on my own experience as a Chinese woman, I began to recognise how sociocultural factors had contributed to my disordered eating.

### The importance of women’s appearance in Chinese society

I start with women’s appearance because it is the persistent pressure I feel acutely in my everyday life. In the Chinese context, appearance refers to a holistic concept that includes both the face and the body, perhaps best captured by the widely circulated expression of being “White, young, and slim” (白幼瘦, bái yòu shòu) [22]. This ideal manifested in my own life when anxiety over my double chin in high school triggered my initial weight loss. Moreover, in Chinese society, the favourable thin and oval face is not just a beauty standard; facial appearance in Chinese culture is often imbued with moral and gendered significance and is thought to influence one’s destiny [31].

In Vu-Augier de Montgrémier et al.’s research, I stated how a woman’s good appearance can give her an advantage in job interviews [19]. To this day, though I despise the practice of incorporating women’s looks into job qualifications, I have to resign myself to it. “Good appearance and temperament” (形象好气质佳, xíng xiàng hǎo qì zhì jiā) is a silent requirement many Chinese employers impose when hiring female employees. I still feel, with shame, that I need to maintain a slender figure at least until I secure a job. A good appearance also increases the likelihood of finding a financially capable partner for heterosexual women. I hate admitting this because it builds upon me, as a woman myself, objectifying women through the male gaze. Yet, few would deny that many Chinese men prefer physically attractive wives. Considering the stakes, both in employment and heterosexual relationships, I agree with Leung et al. that thinness is a highly valued quality among Chinese women [32]. The internalisation of such values leads many to see thinness as integral to their self-worth and a form of social obligation [19].

Body dissatisfaction and thinness preferences among Chinese women are deeply reinforced by social media and the fashion industry [4, 24]. I have lost count of how many weight-related trending topics I have seen on Weibo, how many celebrities have shared their dieting tips, how many before-and-after weight-loss photos included captions like “You’ll never know how beautiful you are until you lose weight” (不瘦下来你永远不知道自己有多美, bù shòu xià lái nǐ yǒng yuǎn bù zhī dào zì jǐ yǒu duō měi) have bumped to me on Xiaohongshu, and how many size-L trousers in Chinese stores I couldn’t fit into. These messages reinforce the notion that only thin women are beautiful, acceptable, desirable, and good, deepening women’s body dissatisfaction and heightening their vulnerability to eating disorders. While Pirie notes that how individuals interpret media images depends on many factors such as their friends and family [4], how could I avoid internalising the ideal of thinness in an environment where it is so strongly privileged. I simply cannot.

### The competition culture within Chinese society

Vu-Augier de Montgrémier et al. note that academic pressure is a trigger for eating disorders among some of their interviewees [19]. I believe this academic pressure stems from a broader culture of competition in China. As a Chinese youth who came of age within a highly competitive educational system, I experienced intense pressure that, to me, mirrored a form of social Darwinism — a constant feeling of being in a race where falling behind means failure. Kipnis observes that Chinese parents often worry about their children falling behind and constantly push them to outperform others in all aspects of “quality” (素质, sù zhì) [33]. Promoted by the government, “quality” is an evaluative discourse encompassing “innate and nurtured physical, intellectual and ideological characteristics of a person” [34]. In China, being “high quality” equates to being good, and parents expect their children to embody this ideal.

These expectations are especially intense in “one-child families,” a legacy of China’s population policy from the 1980 s to the early 2000s. Without siblings, the only child bears “intense pressure to achieve success, good marriages, and better economic standing” [31]. As an only child, I have always felt that my parents’ love is conditional upon me surpassing others and meeting their expectations [19], though my parents strongly deny it. To be fair, their anxiety is understandable, as China’s large population and the highly competitive examination regime offer little room for error [35].

The competitive ideology is further disseminated through state subtle infiltration on scientific child-rearing, embedded in newspapers, television programs, and public billboards [36]. As Greenhalgh explains, “the state’s new norms for child health and education were eagerly, indeed anxiously, taken up by parents” [37] who desperately want to ensure their child not only survived but thrived in a rapidly changing society. Children raised in such a culture are thus pressured to apply the same self-discipline to their bodies as they do to academics.

### Confucian gender norms

To be clear, I do not intend to reject Confucianism in its entirety. However, from a feminist standpoint, I argue that Confucian culture subtly contributes to the development of eating disorders among Chinese women.

In China, “Confucianism endorsed male superiority and emphasized women’s submission and subordination” [22]. While patriarchy is a global phenomenon, Confucianism supplies the specific moral and familial vocabulary through which gender hierarchy is legitimised and reproduced in China. Concepts such as womanly appearance and demeanor (妇容, fùróng) propel women to internalise self-discipline as moral virtue. Thus, rather than being a generic patriarchal influence, Confucianism operates as a special cultural logic that transmutes women’s bodily restraint and submissiveness from socially acceptable behaviours into morally commendable virtues.

As this cultural logic becomes deeply internalised, many women, whether or not they suffer from eating disorders, lack a strong sense of self, and (often unconsciously) objectify themselves through the male gaze. In this sense, the urge to be thin reflects an internalised form of male aesthetic discipline. I remember once believing that I needed to be thin simply to avoid being judged by men. Looking back, I can see how ridiculous I was: I will always be judged regardless of how I look. That said, I do not think women like me in the past should be blamed for conforming to male aesthetics. We are also victims of the male gaze and structural gender inequality. As Saunders et al. put it, we “assimilate to an observer-oriented self-perception. as an anticipatory strategy that allows for a modicum of agency in how one will be regarded by others” [38].

In my view, women’s intense concern with body image reflects a broader societal failure to provide them with security. This too stems from the Confucian ideology, specifically the gendered division of roles captured in the proverb “take charge of the external affairs, women are responsible for the domestic sphere” (男主外女主内, nán zhǔ wài, nǚ zhǔ nèi). Such traditional norms place modern women in an ambivalent position: they are expected to participate in the workforce, yet stark gender inequalities persist [22]. Even when women possess equal abilities, they are often subject to gender discrimination, such as hiring preference and unequal rewards. Such discrimination makes women difficult to (trust that they can) obtain sufficient economic resources to support their independence, and this, reinforces their belief that they need to rely on men. As a result, many women continue to cater to men’s aesthetic preferences: a submissive, delicate, and controllable wife figure. While some Chinese women are aware of their disempowered position, few are willing to challenge the structure — possibly because they have never been empowered to begin with.

Being lower on the power hierarchy means that many Chinese women lack full control over their life trajectories. I recall how my mother gave up her career to raise me. Citing Chernin’s argument that anorexia “must be placed in relation to [a]… fateful encounter between a mother whose life has not been fulfilled and a daughter now presented with the opportunity for fulfilment” [39], Holmes expressed how she knew herself not wanting to be or have a marriage like her mother was part of her anorexia story [16]. I too feel the same way. Whenever my mother reminded me of her sacrifices, I felt guilt, not gratitude, and a strong desire to avoid becoming like her. Every bite I take today carries a double fear: that I will balloon to the point that I become disfavoured in the job market and lose my independence, or that I will not be physically strong enough to resist the patriarchal system that traps women in the family.

I dislike how overinvolved my mother is in my life, especially when my father remains largely indifferent. I have come to understand that my strict control over food and body is, in many ways, a form of projection in which struggles for freedom and autonomy play out through my eating habits. I remember feeling devastated when my therapist once remarked that many people with mental disorders come from similar family configurations. Perhaps this is why I long for a life that “did not pivot upon heterosexuality” [16].

## Sociocultural factors influencing eating disorders recovery

Most of my treatment for eating disorders took place in China. I was hospitalised twice at the Shanghai Mental Health Center, where I received medication, regular diet therapy, group therapy, cognitive behavioural therapy (CBT), and dialectical behaviour therapy (DBT). After discharge, I established a long-term counselling relationship with a young humanistic therapist. While he offered me unconditional acceptance and facilitated my self-exploration, we were both disappointed by the lack of substantial progress in my eating disorder. Hence, when I came to the UK for my Master’s studies, I sought a new therapist and began a different treatment approach: Enhanced Cognitive Behavioural Therapy (CBTe). Having received treatment in both countries, I became increasingly aware of the sociocultural challenges that Chinese patients face in the process of recovery.

### Scarce and expensive psychiatric resources in China

According to Yang, very few doctors in China are trained to treat eating disorders [31]. This reflects my own experience. Specialists in this area are extremely rare and are concentrated almost exclusively in major cities such as Beijing and Shanghai. For patients living elsewhere, geographic distance is a major barrier to diagnosis and treatment. As Zhang notes, non-resident patients often need to pay additional fees for long-distance transportation and accommodation in big cities [40]. I remember clearly that during my outpatient treatment, I had to travel to Shanghai the day before my appointment and stay in a nearby hotel, just to attend a check-up that lasted less than ten minutes. I also recall that, unlike many other inpatients who received family therapy, my parents were unable to attend any sessions due to the long journey.

In addition to the lack of eating disorder specialists, trained counsellors are also scarce and predominantly located in large urban centres. Before the COVID-19 pandemic, there was some time that I did not seek any counselling because I could not find a therapist qualified in eating disorder treatment who was willing to offer online sessions. I hesitated to bear the cost and logistical burden of regular travel to Shanghai for just one hour of therapy. It was only after the pandemic that I established contact with my humanistic counsellor based in Shanghai, as online therapy became more widely accessible.

The high cost of eating disorder treatment poses another significant challenge to recovery. While Yang notes that eating disorders often affect urban, well-educated women from relatively privileged backgrounds [31], my observation is that many women from modest economic circumstances also suffer from eating disorders. At the Shanghai Mental Health Center, eating disorder patients are hospitalised alongside individuals with depression, anxiety, and obsessive-compulsive disorder. However, the cost of treatment for eating disorders is two to three times higher, in part because hospital stays are typically longer for eating disorder patients [41]. Moreover, patients without registered permanent Shanghai residency (hukou) are not eligible for medical insurance reimbursement. My mother complained about this injustice countless times, yet could only lament her inability to secure a Shanghai hukou for me. Outpatient counselling is also expensive. As Zhang explains, China’s insurance industry does not cover long-term psychotherapy, and specialised therapy for eating disorders is particularly costly [40].

These resource barriers in China stand in stark contrast to my treatment experience in the UK, where, apart from the cost of medication, all clinical visits and therapy sessions are covered by the National Health Service (NHS). Moreover, there are significantly more doctors and therapists trained in eating disorders. The UK government has also demonstrated a stronger commitment to this population, as reflected in “the implementation of an England-wide whole-team training to support the creation of a network of over 70 dedicated community-based eating disorder services for children and young people” [41].

### Neglected trauma in Chinese treatment paradigm

Beyond differences in resource availability, my treatment in the UK also made me realise how the Chinese treatment model often neglects individuals’ underlying psychological trauma. Thompson states that women of colour frequently link their eating problems to a range of traumas [15]. I too have often felt that my refusal to eat or my compulsion to binge, is from my aching soul.

After reaching adulthood, I experienced a traumatic event that left me unable to fully trust anyone. The trauma I carry is an intimate, silent history — not a collective memory but a private rupture. On many nights, I binged in an attempt to soothe my pain, while worrying that weight gain would make me less competitive in society and thus less able to protect myself. While my English therapist saw this personal trauma as central to my disordered eating over the years, my Chinese therapist rarely made such connections. The goal of psychotherapy and hospitalisation in China is often to help patients “function normally,” encouraging those who deviate from the mainstream conform to normality and reintegration into society. This is understandable, as Tu argues, “under the current ‘body-centered’ paradigm, Chinese hospitals are ill-equipped to heal these mental scars” [42]. Furthermore, as Zhang notes, Chinese patients are indeed more inclined to seek advice from medical authorities rather than relational or exploratory therapy [40]. This may reflect a broader clinical tendency in which patients are viewed as subjects requiring correction, rather than as individuals in need of holistic care [42].

Like the hospitals, my parents also tended to focus on my behaviours while ignoring the trauma and emotions underlying them. I once voiced this frustration to my Chinese therapist, who gently reminded me that many people from my parents’ generation, those born in the 1960 s and 1970 s, lacked the resources or cultural framework to engage with emotions. Having grown up in an era of material scarcity and rapid economic transition, emotional needs were often suppressed in favour of survival and productivity. As a result, they learned to endure emotional pain in silence, and expect their children to do the same.

### Stigma and stereotypes

Another major challenge to recovery in China is the pervasive stigma and stereotypes surrounding mental illness. Like other mental illnesses, eating disorders are highly stigmatised in China. According to Yang, “the stigma associated with mental illness leads many Chinese with eating disorders to postpone treatment for as long as possible” [31]. I remember vividly how resistant I was to the idea of visiting a psychiatric hospital for the first time. Decades ago in China, mental illness was deeply stigmatised; it was widely perceived not only as an abnormality but as a moral failure — a blameworthy lapse in personal morality that was thought to be under one’s control. Perhaps for this reason, my mother repeatedly reminded me never to tell others that I was taking anti-anxiety and antidepressant medication, fearing it would jeopardise my career or marriage prospects. I understand that she acted out of concern for my future, nevertheless, her reaction illustrates what Yang describes as gendered stigma: in China, women with mental illness face greater scrutiny and social rejection [31]. And I hate it.

I found consolation when my English therapist assured me that mental medications are common, and that taking them does not imply deficiency or failure. There is no shame in seeking help.

Furthermore, in contrast to depression and anxiety, which have received large public awareness through media campaigns, eating disorders remain underrepresented in Chinese public discourse. The lack of education on eating disorders has allowed harmful stereotypes to persist. Like my parents, many people assume anorexics are simply being stubborn and that bulimics lack self-discipline. I have also encountered disbelief that I could have an eating disorder, simply because I am neither underweight nor obese. Those with “non-extreme” body sizes are rendered invisible even within eating disorder communities.

### The cultural history of food, waste, and shame

Another unique sociocultural challenge in rehabilitation arises from China’s historical memory of famine and the moral significance attached to food. Fairburn encourages individuals with BN and BED to practice leaving food on their plates [8]. Yet in Chinese culture, shaped by the mid-twentieth century food scarcity and respect for agricultural labour, failing to finish one’s meal is seen as shameful. As Holmes and Ma note, “purging is also seen as unacceptable because it is understood as wasting food” [22]. I am deeply affected by this moral code. I feel compelled to clean my plate, and if I fail to do so, I am overwhelmed with guilt and shame.

Oxfeld notes that, food for the Chinese is “a potent means of fulfilling obligations to elders, children, and ancestors, and of cementing and reinvigorating social and emotional connections to friends and relatives” [43]. Meals are saturated with meaning. Eating at a family gathering symbolises love and filial piety; refusing food is perceived as a rejection of kinship and care. The Chinese, live in “a world in which obligations and links to others are constantly being reiterated through the medium of food” [43], and in this world, “eating properly” is a normative morality. I cannot count the number of times I was criticised for refusing to be fed at my grandparents’ table, and “struggling to negotiate conflicting imperatives. [I] ended up eating in secret, and alone” [22].

## Reflexive discussion

Eating disorders among Chinese women not only reflect their chaotic relationship with food but also show the broader “chaos” embedded within Chinese society.

### Neoliberal governmentality

Although the state is often considered authoritarian, eating disorders in China reflect the state’s somewhat neoliberal governmentality, which entails “less coercion and a greater degree of ‘self-governance’” [44]. Yang argues that in patriarchal Chinese society, “women’s spaces of interiority, including the heart, subconsciousness, the inner child, and human potential” [31] are governed through the propagation of thinness as a moral and aesthetic ideal, with the tacit endorsement of the government. Judgements about appearance begin in girlhood and persist throughout her womanhood, becoming “seen as everyday, expected and pervasive, but also wounding and painful, with moments or comments often emerging as vivid memories in the narrating of food/body distress” [22].

As such normative discourses are (unconsciously) internalised, a woman’s body becomes a site of constant surveillance and self-discipline. The internalisation and even active promotion of these ideals by women themselves constitutes a subtle form of self-oppression and coercion [24]. Because no tangible authority visibly enforces this self-policing, these practices appear “natural,” making it more difficult to identify, resist, and change them [24]. This logic aligns with Foucault’s notion of biopolitics, wherein power over life is exercised not through coercion but through the shaping of norms and desires [45]. In this way, the “view of the self by the self” blends with both the imagined and real social norms [16], and the complex and seemingly personal emotions embedded in the eating disorders are, in fact, deeply social and political [46].

From this perspective, eating disorders may not solely signify submission to patriarchal aesthetic norms, but also function as a form of resistance to the state’s inscription of power onto the body. Murphy argues that “internalising and resisting are not mutually exclusive” [34]. In this vein, women’s bodily discipline might be read as a paradoxical expression of freedom — an attempt to reassert freedom over their own bodies through food regulation, when no other forms of bodily autonomy seem possible. Yet, as Greenhalgh warns, such interpretations risk legitimising eating disorders as self-empowering [37]. For Greenhalgh, “self-governance is not simple autonomy, but rather a kind of regulated freedom in which individual desires come to be aligned with those promoted by states, professional experts, market forces, and other governing agents” [37]; the quest for thinness, then, is not genuine autonomy but a sophisticated form of self-objectification.

### Gender inequality and heteronormativity

Eating disorders also speak to the enduring gender inequalities in Chinese society. While Maoist China briefly advanced gender egalitarianism with the slogan “women hold up half the sky (妇女能顶半边天, fù nǚ néng dǐng bàn biān tiān),” the post-communist era has witnessed a re-entrenchment of gender essentialism [31]. The return of women’s primary duties back to home “has widened the income gap between husbands and wives, lowering women’s status and diminishing their control over their own lives, marriages, and reproductive events” [31], this, further increases women’s sense of insecurity.

This pervasive insecurity is well recognised by Chinese counsellors, who identify a lack of security as detrimental to women’s mental and physical well-being [31]. While many factors contribute to this insecurity, I believe structural gender inequality is a foundational cause. Despite progress in awareness of women’s rights, women and girls are still frequently treated as second-class citizens, deemed unfit for bearing the familial and economic responsibilities traditionally assigned to men [19].

As Kleinman notes, women’s voices and status remain marginalised across elite institutions such as corporations, research centres, universities, and bureaucracies [47]. Even when women achieve access to education and employment, they must “still work extra hard and count on favourable circumstances to secure a valued status” [47]. In this context, even women with equal quality are placed at a disadvantage simply because of their gender. As a result, many women have no choice but to “draw on ‘the rice bowl of youth,’ and parlay their beauty and sexuality into professional success” [47], which again, demands strict bodily discipline to align with dominant aesthetic preferences. Such structural pressure partially explains my own ambivalence toward feminism. Although I aspire to radical advocacy, I have to temper my stance, knowing that overt resistance may provoke backlash from male-dominated institutions and thus constrain my already limited agency.

Sadly, I have observed some Chinese women abandon the idea of competing with men altogether, turning instead to the perfection of femininity itself — a strategy that “benefits men in satisfying their masculine gaze and masculine taste and meeting their preferences for a ‘weak wife’” [31].

My mother, like many women of her generation, embodies the Confucian prioritization of family, believing that women’s futures are inextricably tied to men and marriage. As Evans notes, such maternal expectations in urban China are not merely personal beliefs but constitute a key mechanism in the intergenerational transmission of gender norms, whereby a mother’s sacrifices and convictions form a moral framework through which daughters negotiate their own subjectivities [48].

My relationship with my mother is close, yet I recoil when her everyday practices as a diligent wife unconsciously pass on these heteronormative values. Before studying abroad, I identified myself firmly as heterosexual; now, buried quietly beneath my lived identity, is a demi-sexual, queer spirit. Bordo suggests that thinness and starvation can represent a rejection of femininity [49] through “an escape into a childlike, boyish or defeminised form” [16]. I do not, however, repel my feminine subjectivity. I simply want to be a person whose life does not have to revolve around heterosexuality.

Expectations from my mother are further reinforced by social media, which perpetuates the idea that “attractiveness is something that must be worked upon” [4]. Despite knowing that I am trapped within these reproduced and complicit values, what I resent most is my own timidity in voicing these thoughts. It is within this fraught process of social reproduction that the ambiguous semiology of my eating disorder becomes apparent: it embodies both my internalised fear of and resistance to male power and heteronormative scripts. In this sense, my eating disorder is not merely an illness but a political gesture — painful yet defiant.

### Moral crisis

Lastly, I contend that the increasing prevalence of eating disorders in China signals a deeper moral crisis, rooted in the “conflicts between individualistic values and collective values of both the officially endorsed socialist morality and the Confucian tradition” [43].

As Farquhar et al. argue, “the morals and politics of production and consumption, collective duties and personal desires, can be infinitely extended through the allegorical form of eating” [50]. While the state has attempted to regulate eating through biopolitical mechanisms and discourses of scientific nutrition, so far, it has failed to “offer stable answers to the question of how to eat both ethically and with pleasure” [50].

As mentioned, eating in China is not simply behavioural but moral. During the Mao era, “eating from the big pot” (吃大锅饭, chī dà guō fàn) metaphorised collectivism and egalitarianism [51]. As that commensal eating collapsed, in the Deng era of reform and opening up, indulging lavish meals became a display of personal achievement and status. In the 2000 s — an era of globalisation, a new discourse emerged. Eating shifted “from prioritizing collective well-being and well-being national welfare to focusing on individual happiness and self-growth defined among Chinese by a sense of contentment, fulfillment, and family harmony” [35]. This change has produced “competing socialist-collectivist and neoliberal-individualist orders” in the moral imagination of Chinese people [52].

Hansen and Svarverud argue that “for the current generation of Chinese youth, the primacy of personal happiness and individual realization has without doubt become the ultimate goal in life, indicating that society has undergone an ethical shift from collective-oriented values to individual-oriented values” [53]. While this observation captures an important societal trend, my lived experience as a Chinese youth suggests a more complex reality. I do not think my generation has entirely abandoned Confucian or collectivist values; rather, we are caught in a state of persistent tension between the allure of liberal individualism and the enduring pull of traditional collectivism [19]. It is within this unsettled moral tension that I trace the roots of my OSFED: the sporadic self-assertion of a binge clashes violently with the lingering sense of being governed by a collective moral duty, with my body and appetite becoming the proxy battlefield.

With traditional moral frameworks destabilised and new values lacking firm grounding, young people like me struggle to find a consistent ethical orientation. In such a moral vacuum, it becomes easier to rely on external markers, such as appearance, to define self-worth. From this perspective, my anorexia may be less about control and more about the desperate attempt to impose order on inner chaos. Likewise, my bingeing or purging may be understood as “an undesirable but understandable way to manage these contradictions — of relinquishing control over body and food, of respecting familial food cultures, and the need to display a female body which signified cultural imperatives of self-restraint” [22].

## Conclusion

“The consumption of food is at once the most ordinary of human activities and one fraught with significance. It not only satisfies our bodily needs but also is associated with our concepts of self, group, and even nationality” [36]. Adopting a critical autoethnographic approach, this paper has examined the sociocultural factors associated with the development and recovery of eating disorders among Chinese women. Individual experience suggests that importance of women’s appearance, a culture of competition, and Confucian gender norms make Chinese women particularly vulnerable to eating problems. In the recovery process, recovery is hindered by scarce psychiatric resources, trauma-neglecting treatment paradigms, social stigma and stereotypes, and a cultural history that associates food-wasting with shame. Beyond lived experience, this paper reflects on how women’s eating disorders expose broader issues of neoliberal governmentality, gender inequality, and moral crisis in contemporary Chinese society.

In New Zealand, being mocked for having a “double chin” made me experience firsthand the universality of body shaming. Yet, it was within the specific aesthetic values and fatalistic beliefs of Chinese culture that this shame was internalised into an obsessive pursuit of thinness. This suggests that while the triggers of eating disorders may be globalised, the cultural narratives that feed them and the pain that individuals endure are profoundly local. My time in the UK exposed me to a therapeutic paradigm that encouraged open discussion of trauma and self-compassion. I gained a temporary sense of liberation — a feeling that my voice and suffering were recognised, and from which I could and should begin to fight back. Yet, as a Chinese woman still living with an eating disorder, I am acutely aware of how fragile my subjectivity remains in the Chinese context. The support for recovery that I long for is constrained by China’s limited medical resources and moral expectations. The dissonance between these two worlds made me doubt whether recovery should be directed towards a negotiated balance between self-affirmation and embeddedness within one’s cultural and moral community.

To me, my eating disorder has been an embodiment of the struggles I face in the pursuit of selfhood and freedom. Yet, as Kleinman notes, the self within the Chinese cannot be easily distinguished due to the blur between “what is deeply personal and what is an intrusion of the authoritarian state into speech and behavior” [47]. Similarly, Greenhalgh contends that bodily self-discipline in contemporary China “is not freedom in the sense of simple autonomy. Instead, it is an artifact of ‘practices of liberty’ embedded in systems of domination” [37]. If their arguments hold, I must ask: who am I really? How can I locate my true self? Could my obsessive self-discipline around food be a distorted pursuit of freedom — one I am reluctant to relinquish because it paradoxically empowers me?

Precisely because the self is so entangled with sociopolitical forces, transforming the social conditions that sustain eating disorders becomes paramount. Thompson rightly argues that “the prevention of eating problems depends not simply upon individual healing but also on changing the social conditions that underlie their etiology” [15]. The sociocultural barriers identified in this paper point directly to the need for systemic reforms. These include nationwide anti-stigma campaigns to enhance public understanding of eating disorders, expansion of the professional workforce, decentralisation of specialist care to major regional hubs, and the integration of trauma-informed practices into therapeutic frameworks [54, 55]. Such measures are crucial first steps toward building a society where recovery is not an individual struggle but a collectively supported possibility.

This vision of recovery is enriched by feminist approaches, which opens the door to healing grounded in “engaging with feminist texts, hearing about feminist ideas from clinicians, and forming relationships with female role models” [56]. In that spirit, I propose that rehabilitation of eating disorders in Chinese women must go beyond the restoration of normative eating behaviours; it must also entail psychological and philosophical rebirth — the shaping and reshaping of subjectivity. Nietzsche’s concept of the Übermensch may be illuminating here. For Nietzsche, “Ubermensch is a being who is able to completely affirm life: someone who says ‘yes’ to everything that comes their way; a being who is able to be their own determiner of value; sculpt their characteristics and circumstances into a beautiful, empowered, ecstatic whole; and fulfill their ultimate potential to become who they truly are” [57]. I envision recovery as a path toward such becoming — not merely the cessation of disordered behaviour, but the emergence of a person no longer governed by external judgment, one who reclaims authorship over her self-identity. In this sense, I still hold hope that a renewed sense of agency might one day liberate me — from both my eating disorder and the sociocultural forces that sustain it.

## Data Availability

No datasets were generated or analysed during the current study.

## Abbreviations

AN: Anorexia Nervosa
BN: Bulimia Nervosa
BED: Binge Eating Disorder
CBT: Cognitive Behaviour Therapy
DBT: Dialectical Behaviour Therapy
NHS: National Health Service
OSFED: Other Specified Feeding and Eating disorder
